# Multiscale Statistical Analysis of Massive Corrosion Pits Based on Image Recognition of High Resolution and Large Field-of-View Images

**DOI:** 10.3390/ma13214695

**Published:** 2020-10-22

**Authors:** Yafei Wang, Zhiqiang Tian, Songyan Hu

**Affiliations:** School of Chemical Engineering and Technology, Xi’an Jiaotong University, Xi’an 710049, China; tzq2232613632@stu.xjtu.edu.cn (Z.T.); hu3118316063@stu.xjtu.edu.cn (S.H.)

**Keywords:** high strength steel, pitting corrosion, statistical analysis, spatial distribution, image recognition

## Abstract

In the present study, a new multiscale method is proposed for the statistical analysis of spatial distribution of massive corrosion pits, based on the image recognition of high resolution and large field-of-view (montage) optical images. Pitting corrosion for high strength pipeline steel exposed to sodium chloride solution was observed using an optical microscope. Montage images of the corrosion pits were obtained, with a single image containing a large number of corrosion pits. The diameters and locations of all the pits were determined simultaneously using an image recognition algorithm, followed by statistical analysis of the two-dimensional spatial point pattern. The multiscale spatial distributions of pits were analyzed by dividing the montage image into a number of different windows. The results indicate the clear dependence of distribution features on the spatial scales. The proposed method can provide a better understanding of the pit growth from the perspective of multiscale spatial evolution.

## 1. Introduction

High strength steels are widely utilized to build long-distance oil and gas pipelines to significantly reduce construction costs. However, many pipelines are susceptible to pitting corrosion when they are exposed to different kinds of harsh environments such as chloride, H_2_S and CO_2_ containing solutions [1,2,3]. The failure of pipelines can result in catastrophic failures, leading to severe economic and casualty losses [4,5,6,7,8]. The prevention of corrosion induced failures requires in-depth understanding of the evolution behavior of corrosion pits.

The initiation and propagation mechanisms of a single pit for different structural materials, including high strength pipeline steels, have been extensively studied in the past decades. These studies typically involve electrochemical measurement [9,10], characterization of typical corrosion morphologies [11] and numerical modeling [12]. The past studies in literature reveal that the inclusions in steel play a critical role in the pitting initiation [13,14,15]. Reactions tend to occur preferentially around the inclusions, probably due to the potential difference between the inclusion and the matrix, which was demonstrated by some scholars using advanced surface measurement techniques such as scanning Kelvin probe force microscopy [16,17,18]. In our previous study, corrosion pit features on X80 high strength pipeline steel formed in sodium chloride solution were systematically investigated [19]. Massive corrosion pits with regular shapes were identified on the specimen surface after a short period of immersion tests and the pits were found to grow preferentially in the horizontal direction [20].

Previous studies are mostly focused on the initiation and propagation mechanisms of a single pit or a very small number of pits. The high-resolution morphologies of the corrosion pits are usually presented, from which the related information about different stages of pitting corrosion can be obtained. For localized corrosion such as pitting and crevice corrosion, precise understand of the conditions at every point on a metal surface is unlikely, and statistic techniques thereby become necessary for the quantitative characterization of the corrosion processes. Therefore, many studies are devoted to the study of statistical features of corrosion pits including spatial statistics [21]. However, the microstructures and corrosion processes are usually exhibiting scale-dependent features [22,23] and the high-resolution images with small field-of-view used in the traditional analysis can only provide limited information for typical corrosion pits. The distribution of corrosion pits in two-dimensional space and their evolution with time are currently not well understood. This is partially due to the difficulty of precisely determining the locations of all pits at the same time. In our previous study, an image recognition method named gradient-based Hough transformation was applied to the analysis of corrosion pits, which was proved to be effective for accurate recognition of the pit diameters and locations [24,25]. In this paper, this technique was further applied to the recognition of high-resolution and large field-of-view images and a new multiscale analysis method was proposed, which can reveal the spatial distributions of pits in different scales.

## 2. Material and Methods

### 2.1. Material and Corrosion Tests

The material studied here was X80 high strength pipeline steel (API Spec 5L, Beijing, China), which was provided by the China Special Equipment Inspection and Research Institute. The chemical composition of the steel was (wt.%): C 0.07, Mn 1.77, Ni 0.22, Mo 0.21, Si 0.30, P 0.02, S 0.005, Cu 0.22 and the balance was Fe. Immersion tests were performed to obtain the corrosion morphologies. Specimens with dimensions of 10 mm × 10 mm × 5 mm were cut from the steel plate with a thickness of 15 mm. Then, they were ground with 400–1200 grid SiC abrasive papers gradually, mechanically polished with 1 μm diamond paste and washed with distilled water and acetone (Sinopharm Chemical Reagent Co., Ltd., Shanghai, China). The specimens were immersed in a 3.5 w% NaCl solution for 1 h and then immediately prepared for microscopic observations. The morphologies of the corrosion pits on the top surface of the specimens with an area of 10 mm × 10 mm were observed under an optical microscope (Opto-digital DSX500, Olympus, Tokyo, Japan). The entire morphology of the surface was obtained through one single optical image, in other words, the high-resolution and large field-of-view image, by precisely stitching 16 small images precisely together.

### 2.2. Image-Recognition Method

Sizes and locations of pits in the corrosion image were obtained using the image recognition algorithm in MATLAB software. The image recognition method used here was originally proposed by Peng et al. [26] and applied to the analysis of corrosion images in our previous study [24]. This method is based on the Hough transform [27,28], and its key concept is described as follows. The circle in a two-dimensional plane can be expressed by:

(1) $${\left(x-a\right)}^{2}+{\left(y-b\right)}^{2}=r^{2}$$

In Equation (1), (*a*, *b*) is the coordinate of the center of the circle, and *r* is the radius of the circle. In the three-parameter (*a*, *b*, *r*) orthogonal coordinate system, the circle in Equation (1) is transformed into a cone, as described in Equation (2).

(2) $${\left(a-x\right)}^{2}+{\left(b-y\right)}^{2}=r^{2}$$

Therefore, all the circles passing through $\left(x_{i},y_{i}\right)$ can be described by Equation (3):

(3) $${\left(a_{i}-x_{i}\right)}^{2}+{\left(b_{i}-y_{i}\right)}^{2}={r_{i}}^{2}$$

Similarly, all parametric equations of circles passing through $\left(x_{j},y_{j}\right)$ can be expressed as:

(4) $${\left(a_{j}-x_{j}\right)}^{2}+{\left(b_{j}-y_{j}\right)}^{2}={r_{j}}^{2}$$

If ($x_{i}$, $y_{i}$) and $\left(x_{j},y_{j}\right)$ are on the same circle, then there must be an intersection of two cones in the parameter space that satisfies Equation (5):

(5) $$a=a_{i}=a_{j},b=b_{i}=b_{j},r=r_{i}=r_{j}$$

**Figure 1:**
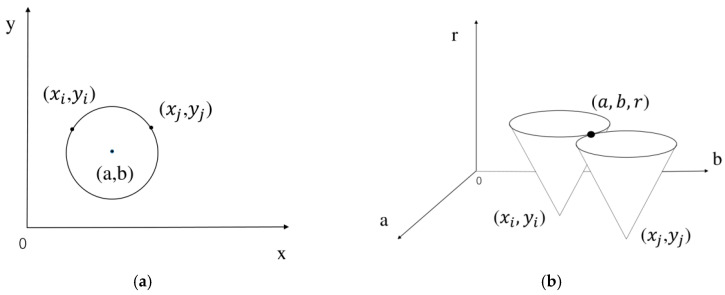
Conversion of a circle in (**a**) two-dimensional space to cones in (**b**) three-dimensional space.

From every intersection, a circle can be obtained. Multiple intersections on the plane correspond to the clusters of cones in the parameter space, as illustrated in Figure 1. Circles can be detected when the range of circle diameters is set. However, the traditional Hough transform method is inefficient in practical applications, so the multidimensional cumulative array method is more commonly used than the parameter transformation method [29]. In a computer, the values of all pixels of the 8-bit grayscale image are between 0 and 255 and a gradient must exist between the pixels in corrosion pits and the surrounding background. The pixel gradient of a point on the edge of a circle should either point to the center of the circle or away from the center, and thereby always lie on a straight line that passes through the center of the circle. A voting process is utilized in order to transform the gradient field into an accumulation array. A weight value is added to the pixels in the accumulation array, which lies on the line segment. The accumulation array can be constructed by collecting the votes from all nonzero gradient vectors. The center of circle can be recognized by finding the maximum intensity in the accumulation array. These recognition processes have been discussed in depth in our previous paper [24].

The original optical image was imported into MATLAB and transformed into a grayscale image. Then, the “filter2” function in the Signal Processing Toolbox was applied to conduct image noise reduction, which can improve the accuracy of detection. Then the parameters were defined, including the range of diameters, the threshold of gradient, et cetera. The size of the montage image was too large (3775 × 3803 pixels) for one-step recognition, so the image was divided into 16 parts and the recognition programs were run separately. The recognized data for the small images were combined together to give an accurate recognition of the whole image.

### 2.3. Multiscale Spatial Point Pattern

The spatial distribution of corrosion pits was analyzed using the Ripley’s function in spatstat package of R language [30], which is recognized as a powerful tool for the analysis of two-dimensional spatial point patterns in many fields [31,32,33]. Ripley’s K and L functions provide intrinsic information about the spatial distributions of multiple points in two-dimensional space, based on the mean value of the point density within a circular area with a radius of space scale *r*, as shown in Equation (6).

(6) $$k\left(r\right)=\frac{A}{N^{2}}\sum_{i=1}^{N}\sum_{j=1}^{N}\frac{I_{r}(d_{ij)}}{\omega_{ij}}\left(i\neq j\right)$$

In this equation, *r* is the radius of the circular area of interest. *A* is the area of the whole image, *N* is the number of points in the circular area and $d_{ij}$ is the distance between point i and point *j*. $I_{r}$ is a Boolean function. When $d_{ij}$ > r, $I_{r}$ = 0; when $d_{ij}$ < r, $I_{r}$ = 1. $\omega_{ij}$ is the edge correction coefficient. Ripley’s L function, which is a different form of K function, is more commonly used, as shown in Equation (7).

(7) $$L\left(r\right)=\sqrt{\frac{K\left(r\right)}{\pi }}-r$$

**Figure 2:**
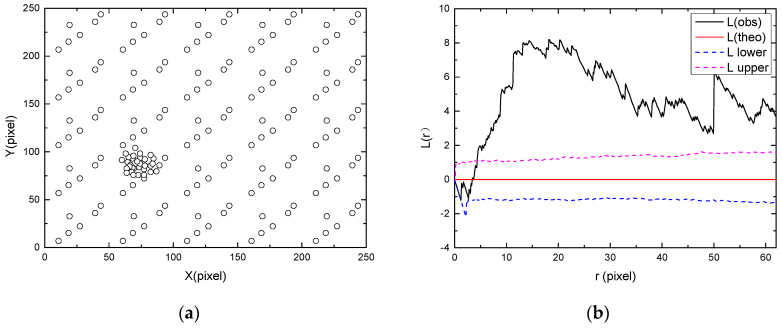

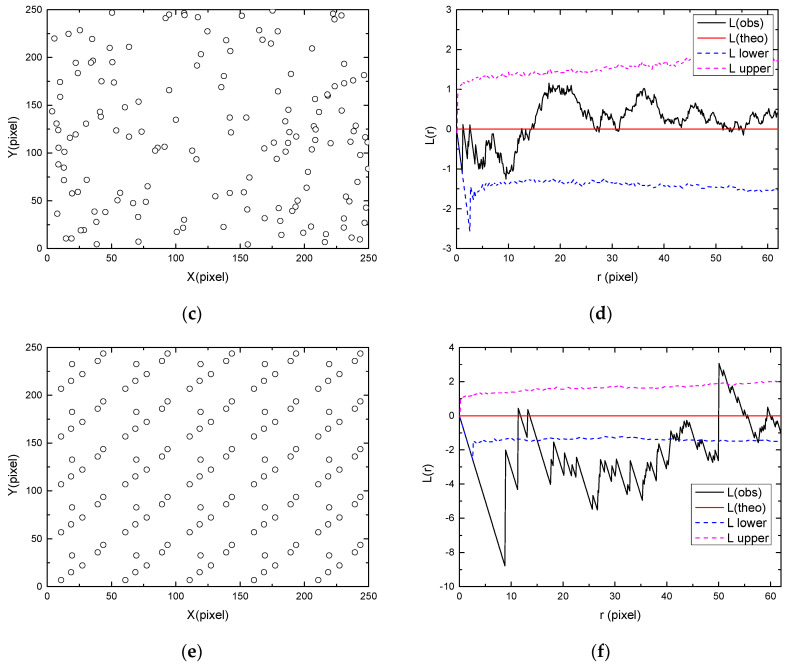
(**a**) Clustered, (**c**) random and (**e**) regular point patterns, with the corresponding values of L functions shown in (**b**), (**d**) and (**f**), respectively. “L(theo)” is the theoretical value of the L function under the condition of complete spatial randomness, “L(obs)” is the value of L function for the analyzed point pattern, “L upper” and “L lower” are the upper and lower bounds with 95% confidence, which were generated by Monte Carlo simulation [39].

The spatial distributions of point patterns can be classified into three categories based on the values of Ripley’s L function [34]: clustered, random and regular patterns, as illustrated in Figure 2. If most of the L function values are larger than the upper bound, the points can be considered as a clustered pattern (Figure 2a). If the values of the L function are always between the upper and lower bounds, the spatial randomness of points can be confirmed (Figure 2b). If most of the L function values are smaller than the lower bound, it can be considered that the points are regularly distributed (Figure 2c). In the present study, the effect of window size on the spatial distribution was also studied. The montage image was divided into different numbers of small regions such as 1 × 1 (original image), 2 × 2, 3 × 3 and 4 × 4. Then, Ripley’s L function was calculated for each region to obtain multiscale information on the pit distribution. In the study of multiscale spatial point patterns, as previously discussed by Liu et al. [35] and some others, two kinds of scales are usually defined: one is the scale of data and the other is the scale of analysis. In the present study, the scale of data is the size of observation window (944 × 951, 1258 × 1268, 1888 × 1902 and 3775 × 3803 pixels), and the scale of analysis is the radius of circle (*r*) in the calculation of L function. Traditional analysis on pit distributions only involves the scale of analysis [36,37,38] and the spatial distributions in a very small region (in the scale of micrometers) are usually investigated in these studies. The multiscale analysis in our paper is focused on a large field-of-view image with a larger scale of data than those used in conventional analyses, which enables revealing of the distribution features at larger scales.

## 3. Results and Discussion

### 3.1. Image Recognition

**Figure 3:**
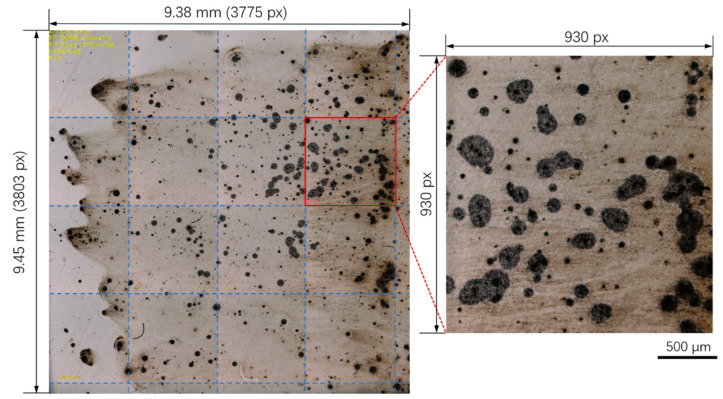
The high-resolution and large field-of-view image of corrosion pits formed on X80 pipeline steel after immersion in 3.5 w% NaCl solution for 30 min, with the enlarged image shown on the right.

The high resolution and large field-of-view image was obtained on the top surface of the specimen with a size of 10 mm × 10 mm after immersion tests, as presented in Figure 3. The edges of the specimen were affected by the mechanical grinding and polishing processes. Thus, only the majority of the specimen surface was analyzed with an area of approximately 9.38 × 9.45 mm. This montage image consisted of 16 small images as indicated by the dashed lines, with one of them shown on the right. The size of each small image was about 930 × 930 pixels and the size of the whole image was 3775 × 3803 pixels. Severe pitting corrosion was observed and most of the pits exhibited regular round shapes. As already demonstrated in our previous studies, the circular corrosion pits were initiated from spherical Al-Ca-O-S type inclusions [13]. Hundreds of pits were identified in Figure 3 with different sizes, implying that the pits were initiated at different times. The pits with small sizes, in other words, the “young” pits, were likely initiated after the “old” pits.

**Figure 4:**
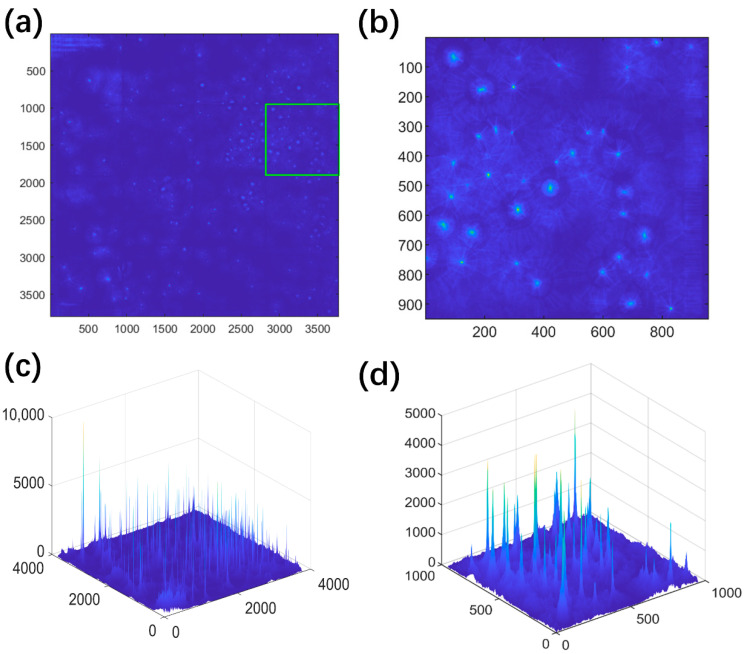
Two-dimensional accumulation arrays of Figure 3 obtained by image-recognition method [26] in MATLAB for (**a**) whole image and (**b**) enlarged image, with the corresponding three-dimensional accumulation arrays shown in (**c**) and (**d**). Local peaks in the 2D and 3D accumulation arrays correspond to the locations of pits.

**Figure 5:**
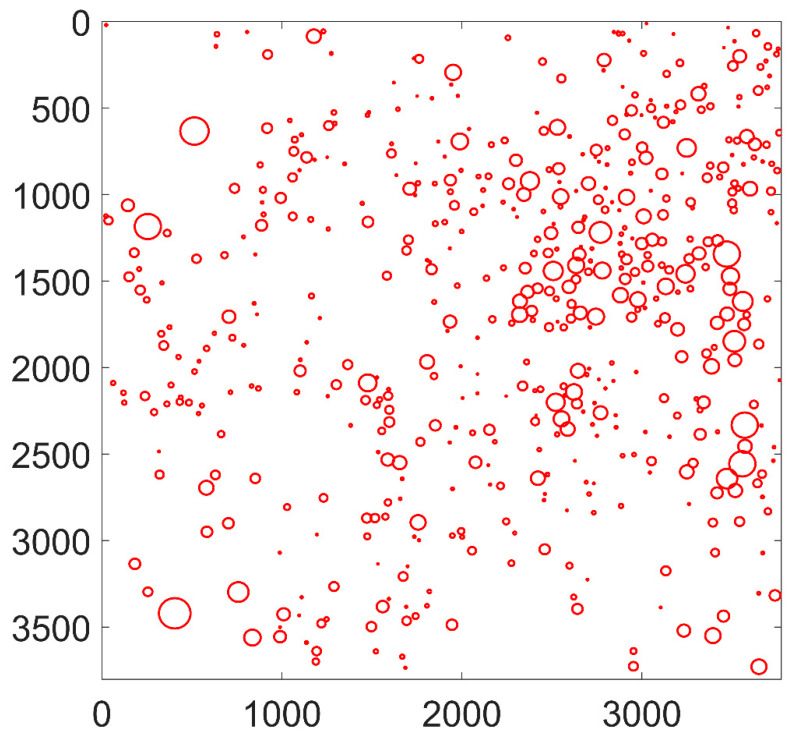
Pits identified from the original montage image shown in Figure 3 with a total number of 532.

To quantify the spatial distribution of the massive pits, the pit locations were obtained through the Hough transform algorithm, as shown in Figure 4 and Figure 5. The two-dimensional and three-dimensional accumulation arrays obtained through the Hough transform method are shown in Figure 4. The local peaks corresponding to the locations of pits can be clearly identified in the arrays, as discussed in the previous section. The corrosion pits identified in Figure 3 are replotted in Figure 5 with a total number of 532. The radii of pits were found to be in the range of 4 to 88 pixels. Our previous study demonstrated through numerous tests that the diameters of pits follow the lognormal distribution [25]. The present study focused on the pit locations while the information about pit radii was ignored.

### 3.2. Multiscale Spatial Distribution

**Figure 6:**
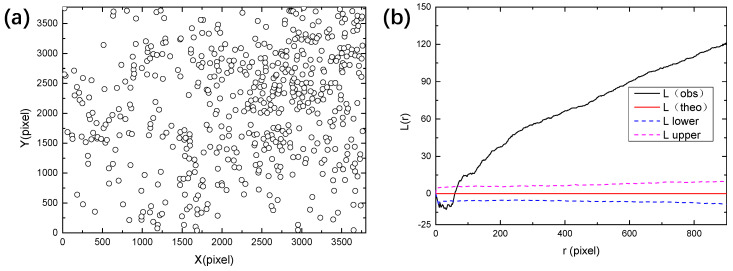
(**a**) Recognized pit locations from Figure 4 and (**b**) Ripley’s L function for pits calculated by the spatstat package of R language [30]. “L(theo)” is the theoretical value of the L function under the condition of complete spatial randomness, “L(obs)” is the value of L function for the analyzed point pattern, “L upper” and “L lower” are the upper and lower bounds with 95% confidence, which were generated by Monte Carlo simulation. The values of L function in (b) are consistent with those of the “clustered” pattern shown in Figure 2.

The pit locations and Ripley’s L function for the whole montage image are shown in Figure 6. As from Figure 6b, the values of L function are much higher than the upper bound for most *r* values, indicating a clustered distribution. This agrees with the spatial pattern shown in Figure 6a, which indicates that the majority of pits are located in the upper right region of the specimen while the pit densities in the other regions are much smaller.

**Figure 7:**
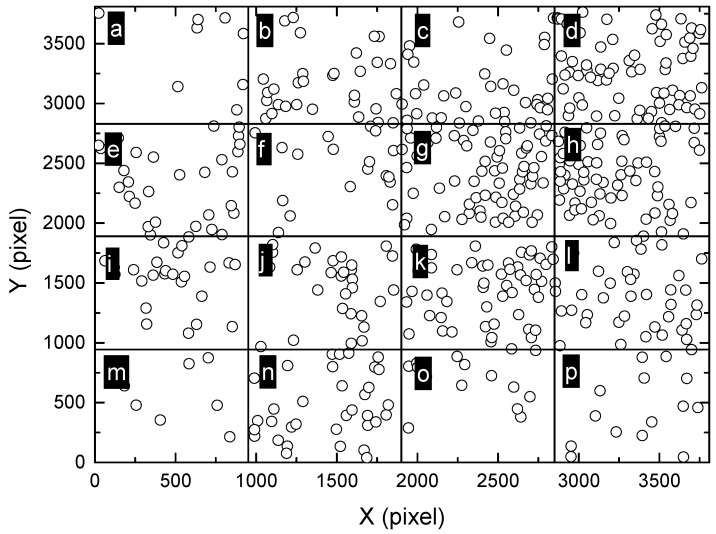
Division of the montage image (Figure 6a) into 4 × 4 sub-regions, marked as (**a**–**p**). The size of each sub-region is approximately 944 × 951 pixels, which is amongst the most commonly used field-of-view in traditional microscopic observations, corresponding to the smallest scale of observation in the present study.

**Figure 8:**
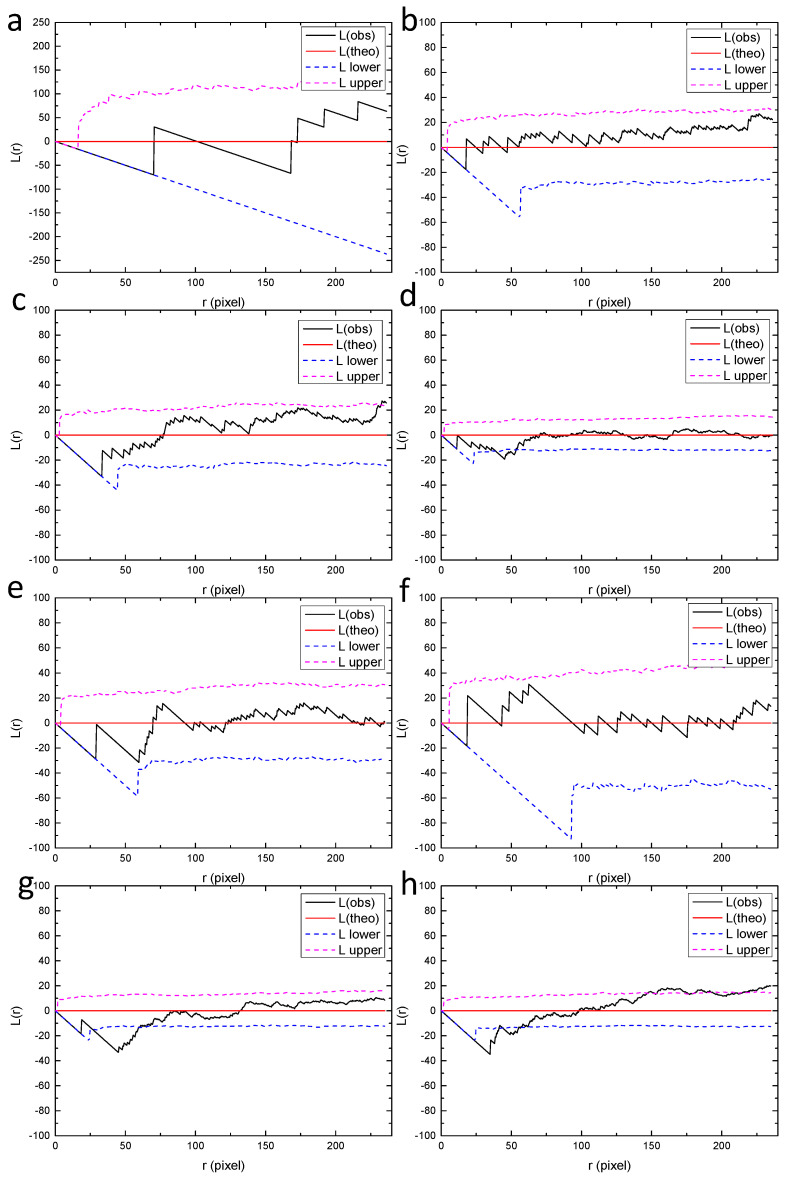

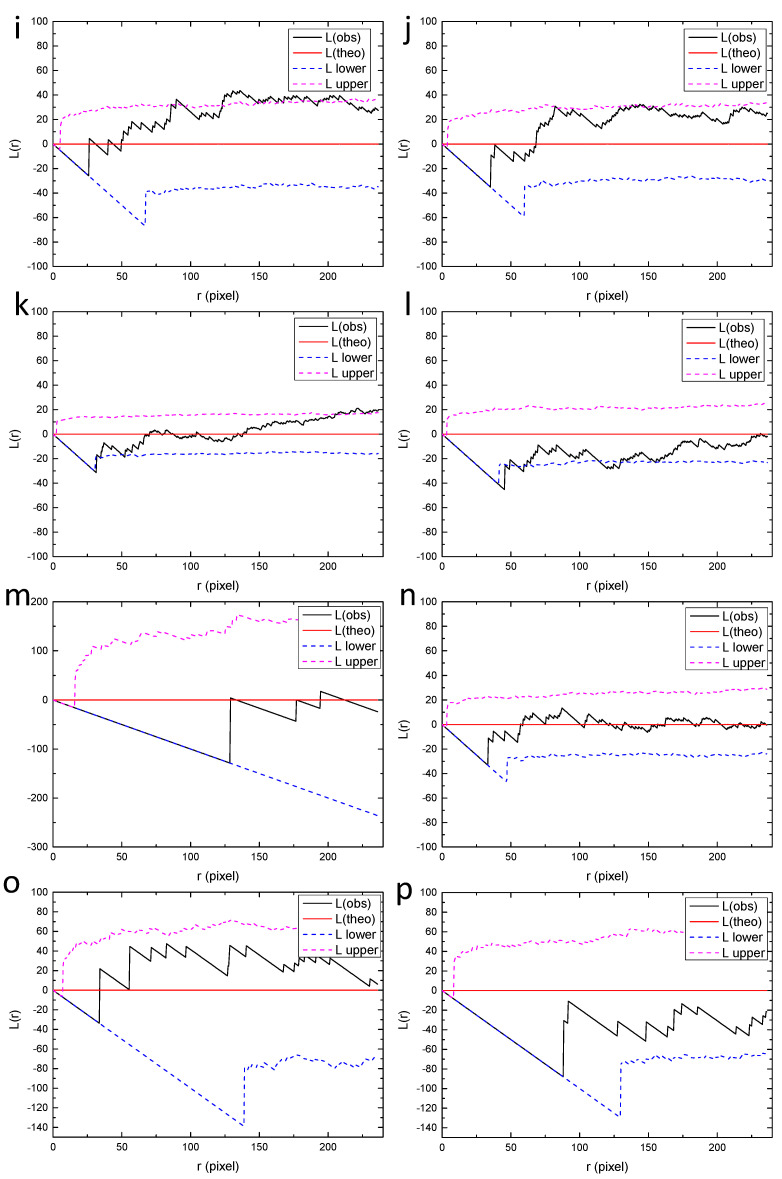
Values of L function for the small regions from (**a**–**p**) in Figure 7, calculated by the spatstat package of R language. “L(theo)” is the theoretical value of the L function under the condition of complete spatial randomness, “L(obs)” is the value of L function for the analyzed point pattern, “L upper” and “L lower” are the upper and lower bounds with 95% confidence, which were generated by Monte Carlo simulation.

In order to further reveal the distribution characteristics of massive pits at different scales, the original montage image shown in Figure 6a was divided into different smaller sub-regions (2 × 2, 3 × 3 and 4 × 4). Ripley’s L function was applied to these small regions separately. The smaller the sub-regions are, the smaller is the scale of observation. Firstly, the montage image was divided into 16 rectangular sub-regions, with each sub-region having a size of 944 × 951 pixels, as shown in Figure 7. The size of the sub-region is one of the most commonly used field-of-view in traditional microscopic observations, corresponding to the smallest scale of observation in the present study. Then, the values of L function were calculated for these regions from “a” to “p”, as shown in Figure 8. It can be seen that most of the regions exhibit random distribution, such as “a” to “f”. For these regions, the values of L function lie between the upper and lower bounds, identical to the case of complete spatial randomness, as shown in Figure 2b. It is worth noting that actual patterns are never completely spatially random, in other words, the Poisson process patterns. The curves of L function for “a” to “f” only imply that these patterns are close to a Poisson process sample [40]. For some other regions such as “h”, “i” and “k”, the values of L function tend to increase with *r* and become slightly higher than the upper bound when *r* is sufficiently large. This indicates the tendency of clustering at larger scales. However, the deviation from the bounds of random patterns is insignificant. Therefore, all the patterns in Figure 7 including “h”, “i” and “k” are considered to be close to random distributions.

**Figure 9:**
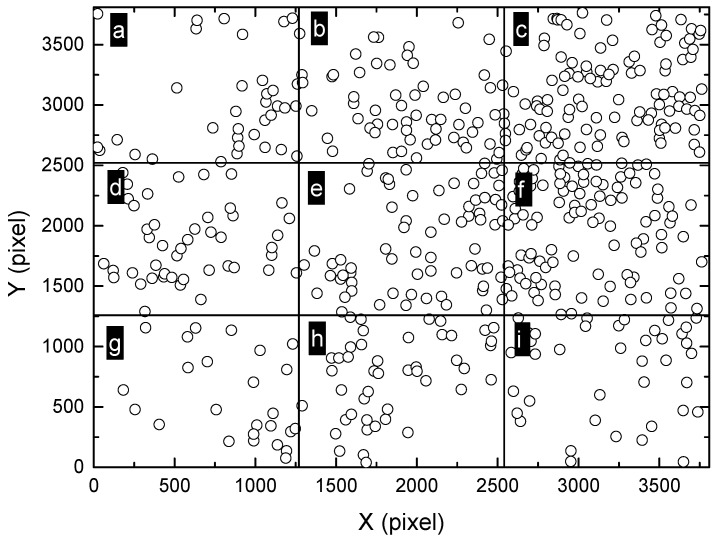
Division of the montage image (Figure 6a) into 3 × 3 sub-regions, marked as (**a**–**i**). The size of each sub-region is approximately 1258 × 1268 pixels.

**Figure 10:**
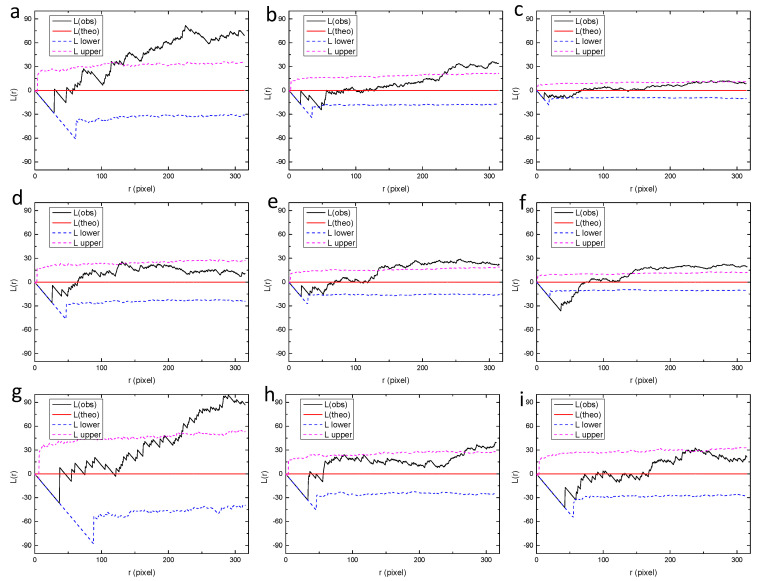
Ripley’s L function for the sub-regions from (**a**–**i**) in Figure 9, calculated by the spatstat package of R language. “L(theo)” is the theoretical value of the L function under the condition of complete spatial randomness, “L(obs)” is the value of L function for the analyzed point pattern, “L upper” and “L lower” are the upper and lower bounds with 95% confidence, which were generated by Monte Carlo simulation.

Furthermore, a larger scale was considered and the field-of-view was zoomed out, corresponding to 3 × 3 sub-regions with the size of 1258 × 1268 pixels, as shown in Figure 9. The corresponding L functions are shown in Figure 10. Interestingly, some of the regions change from random distribution to clustered distribution, such as “a”, “b”, “e”, “f” and “g”. Other regions marked as “c”, “d”, “h” and “i” are identified as random patterns. These observations are consistent with the point patterns in Figure 9. Some patterns are very similar, such as the clustered pattern “e” and the random pattern “d”, although there is a clear difference in their corresponding L functions.

**Figure 11:**
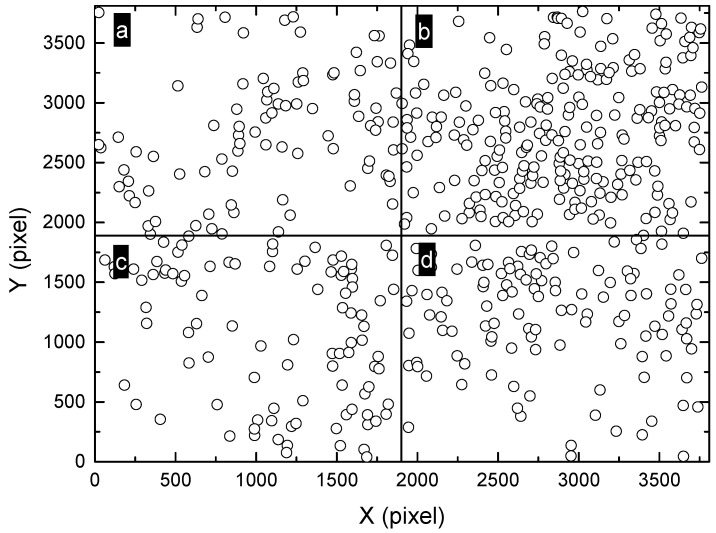
Division of the montage image (Figure 6a) into 2 × 2 sub-regions, marked as (**a**–**d**). The size of each sub-region is approximately 1888 × 1902 pixels.

**Figure 12:**
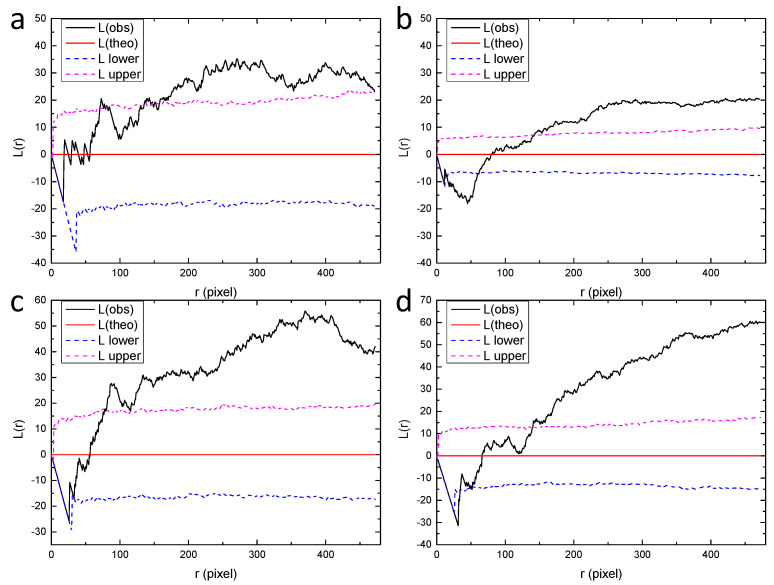
Ripley’s L function for the sub-regions (**a**–**d**) in Figure 11, calculated by the spatstat package of R language. “L(theo)” is the theoretical value of the L function under the condition of complete spatial randomness, “L(obs)” is the value of L function for the analyzed point pattern, “L upper” and “L lower” are the upper and lower bounds with 95% confidence, which were generated by Monte Carlo simulation.

The montage was further divided into larger sub-regions, as shown in Figure 11, whose L functions are shown in Figure 12. It is clearly observed that all sub-regions exhibit apparently clustered patterns, with a large deviation from random distribution. This implies that the extent of point aggregation tends to increase with the observation scale.

**Figure 13:**
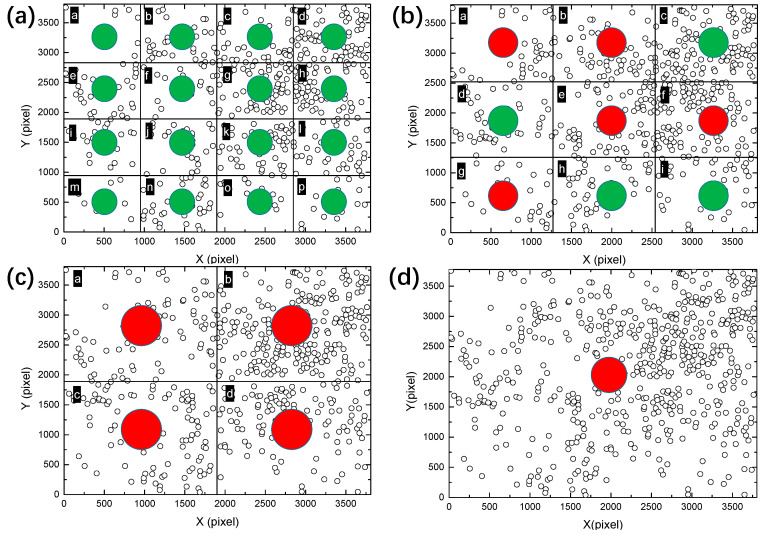
Spatial point patterns identified by Ripley’s L function for the sub-regions of the high resolution and large field-of-view image at different scales by dividing the montage image (Figure 6a) into (**a**) 16, (**b**) 9, (**c**) 4 and (**d**) 1 sub-regions. The green color indicates random distribution and the red color indicates clustered distribution. The clustering of patterns is progressively lost when the scale of observation is decreased, highlighting the advantage of using large field-of-view images to reveal scale-dependent spatial distributions.

Overall, Figure 13 shows the type of point patterns identified by Ripley’s L function for the high resolution and large field-of-view image at different scales. It is observed that the fraction of clustered patterns among all the sub-regions tend to increase gradually with the scale of observation. For the smallest scale of 4 × 4 regions, no patterns are clustered and all of them are close to random patterns. For a larger scale of 3 × 3 regions, more than half of the regions are clustered patterns. For the 2 × 2 and 1 × 1 regions, all the patterns are clustered. This indicates a strong dependence of the spatial point pattern on the scale of observation. The randomly distributed corrosion pits observed in the sub-regions in Figure 13a, are actually part of the clustered patterns at larger scale, as shown in Figure 13d. Since the resolution of the sub-region in Figure 13a is close to that commonly used in traditional microscopic observations, it is thereby concluded that the traditional small field-of-view image can only provide very limited information of the corrosion process in the whole specimen. It should be noted that the results in Figure 13 cannot be easily discovered using traditional observation methods such as optical microscope and scanning electron microscope, since the field-of-view of these methods is too small to get the whole distribution features. The application of the multiscale method to the analysis of corrosion pit distribution in large field-of-view images, aided by the automatic image scanning and stitching as well as image recognition (pit locations cannot be easily obtained using traditional methods), should contribute to the more in-depth quantitative understanding of multiscale spatial distributions of pits

This paper presents a new method for the analysis of spatial distributions of corrosion pits at multiple scales, which is theoretically applicable for any material at all testing conditions, as long as the image recognition process performs well. Regular shaped corrosion pits are considered here, for which the Hough transform algorithm shows excellent accuracy and efficiency. For other corrosion pits with irregular shapes, the image recognition process algorithm needs to be updated. For example, the corroded area can be transformed into an equivalent circle and then processed using the Hough transform method, which has been discussed in our previous work [24].

Furthermore, the spatial distributions of massive corrosion pits are the result of corrosion reactions. Therefore, the features of multiscale distribution are dependent on the corrosion mechanisms and thereby on the pitting distributions. The in-depth investigation of corrosion mechanisms is crucial to understanding the multiscale spatial evolution behavior of corrosion pits, which depends on many influencing factors such as ion diffusion, mass transfer, material microstructures, et cetera. Several groups of tests were conducted in the present study, but only one group of the tests is presented here since this image is perfect for image recognition and to show the evolution behavior of pits. For other images, the pits were either not initiated or grew too fast so that the image recognition performed poorly due to the coverage of too much corrosion products. Even though, it is believed that the method introduced here can provide a useful tool for the analysis of scale-dependent pitting distributions and to discover the influencing factors that lead to pitting clusters at different scales. By capturing the multiscale distributions at different times using the method proposed in this study in combination with well-designed experiments for studying corrosion mechanisms, the key factors affecting the pit aggregation behaviors at different scales can be determined, which will improve the understanding of the fast growth process of pits and the resulting failure of structures.

## 4. Conclusions

In the present study, a multiscale analysis method is proposed for the quantitative evaluation of spatial distributions of massive corrosion pits. This method was proved efficient and useful for identifying the type of spatial point patterns of corrosion pits at different scales. Results show that the type of point patterns is strongly dependent on the scale of observation and the traditional small field-of-view images can only provide limited or even misleading information on the whole corrosion process. Specifically, taking a typical montage image as an example, the multiscale analysis showed that the clustering of patterns is progressively lost when the scale of observation is decreased. This highlights the advantage of using large field-of-view images to reveal spatial distributions at multiple scales. Although the multiscale features of point patterns depend on the corrosion mechanisms, the method introduced here is applicable for all materials and testing conditions as long as the locations of pits can be successfully accessed. It can also be applied to images with larger resolutions and sizes, making it possible to reveal the distribution characteristics of corrosion pits over a wider range of scales. By analyzing the multiscale spatial distributions at different immersion times, the multiscale spatial evolution mechanism of corrosion pits may also be revealed.
